# 5-MeO-DMT for post-traumatic stress disorder: a real-world longitudinal case study

**DOI:** 10.3389/fpsyt.2023.1271152

**Published:** 2023-11-23

**Authors:** Anya Ragnhildstveit, Ryan Khan, Paul Seli, Lisa Claire Bass, River Jude August, Miriam Kaiyo, Nathaniel Barr, Laura Kate Jackson, Michael Santo Gaffrey, Joseph Peter Barsuglia, Lynnette Astrid Averill

**Affiliations:** ^1^Integrated Research Literacy Group, Draper, UT, United States; ^2^Department of Psychiatry, University of Cambridge, Cambridge, United Kingdom; ^3^Department of Psychology and Neuroscience, Duke University, Durham, NC, United States; ^4^Neuroscience Interdepartmental Program, University of California, Los Angeles, Los Angeles, CA, United States; ^5^Department of Family and Consumer Studies, University of Utah, Salt Lake City, UT, United States; ^6^School of Humanities and Creativity, Sheridan College, Oakville, ON, Canada; ^7^Department of Pediatrics, Division of Pediatric Psychology and Developmental Medicine, Medical College of Wisconsin, Milwaukee, WI, United States; ^8^Children’s Wisconsin, Milwaukee, WI, United States; ^9^The Mission Within, Baja California, Mexico; ^10^Menninger Department of Psychiatry and Behavioral Sciences, Baylor College of Medicine, Houston, TX, United States; ^11^Michael E. DeBakey Veterans Affairs Medical Center, Houston, TX, United States; ^12^Department of Psychiatry, Yale School of Medicine, New Haven, CT, United States

**Keywords:** 5-methoxy-*N*, *N*-dimethyltryptamine, 5-MeO-DMT, psychedelic therapy, post-traumatic stress disorder, PTSD, trauma, case report

## Abstract

Psychedelic therapy is, arguably, the next frontier in psychiatry. It offers a radical alternative to longstanding, mainstays of treatment, while exciting a paradigm shift in translational science and drug discovery. There is particular interest in 5-methoxy-*N,N*-dimethyltryptamine (5-MeO-DMT)—a serotonergic psychedelic—as a novel, fast-acting therapeutic. Yet, few studies have directly examined 5-MeO-DMT for trauma- or stress-related psychopathology, including post-traumatic stress disorder (PTSD). Herein, we present the first longitudinal case study on 5-MeO-DMT for chronic refractory PTSD, in a 23-year-old female. A single dose of vaporized bufotoxin of the Sonoran Desert Toad (*Incilius alvarius*), containing an estimated 10−15 mg of 5-MeO-DMT, led to clinically significant improvements in PTSD, with next-day effects. This was accompanied by marked reductions in hopelessness and related suicide risk. Improvements, across all constructs, were sustained at 1-, 3-, 6-, and 12-months follow-up, as monitored by a supporting clinician. The subject further endorsed a complete mystical experience, hypothesized to underly 5-MeO-DMT’s therapeutic activity. No drug-related, serious adverse events occurred. Together, results showed that 5-MeO-DMT was generally tolerable, safe to administer, and effective for PTSD; however, this was not without risk. The subject reported acute nausea, overwhelming subjective effects, and late onset of night terrors. Further research is warranted to replicate and extend these findings, which are inherently limited, non-generalizable, and rely on methods not clinically accepted.

## Introduction

5-methoxy-*N,N*-dimethyltryptamine (5-MeO-DMT) is a natural, short-acting psychoactive indolealkylamine (1). It was first synthesized in 1936 (2), later found in several plant (e.g., *Dictyoloma incanescens*), fungi (e.g., *Amanita citrina*), and animal (e.g., *Incilius al*var*ius*) species (2, 3). In humans, 5-MeO-DMT is likely endogenous, with trace amounts detected in urine (2 of 113 people), blood (20 of 39 people), and cerebrospinal fluid (40 of 136 people) (2). However, various studies contradict this finding (2, 4); and its physiological role, if any, remains unclear. Based on ethnographic reports, 5-MeO-DMT may have been used by indigenous cultures, as part of plant extracts and botanical preparations (e.g., *yopo* or *cohoba* snuff), specifically for spiritual and ritualistic practices (5, 6). These reports date back to ancient People of Mesoamerica (5, 6). Yet, there is little evidence to support such claims. Further, contrary to published work, 5-MeO-DMT is not found in traditional or analog ayahuasca (7, 8). This points to its use being a more recent phenomenon (9, 10).

Regarding its pharmacology, 5-MeO-DMT is a nonselective serotonin (5-HT) receptor agonist (11, 12). It also binds to other receptors, including dopamine and serotonin, as well as norepinephrine transporters (12). The entheogen mildly inhibits 5-HT reuptake, yet exerts no appreciable effects on monoamine release (13). 5-MeO-DMT has the highest affinity for 5-HT_1A_ (K_i_, < 10 nM) over 5-HT_2A_ (K_i_, >1,000 nM), with 300–1,000-fold greater selectivity (11, 12, 14). This is notable, given that most serotonergic psychedelics, like LSD and psilocybin, are mediated by 5-HT_2A_ activation (15). Other non-5-HT_2A_ receptors have not been studied as widely (16). Metabolically, 5-MeO-DMT is processed via oxidative deamination—catalyzed by monoamine oxidase A (MAO_A_)—into the active metabolite, bufotenine (17). Use of 5-MeO-DMT with MAO inhibitors (MAOIs), such as antidepressants, can augment and prolong neurochemical and behavioral effects, by blocking biotransformation of 5-MeO-DMT and increasing its exposure (18). Nonetheless, MAOIs can induce serotonergic toxicity (19), or ‘serotonin syndrome’, a potentially life-threatening drug reaction caused by excess serotonin in the brain (20). This can present as shivering or diarrhea, as well as muscle rigidity, high fever, and epileptic seizure. Combining 5-MeO-DMT with harmala alkaloids, short-term MAOIs found in ayahuasca, can also produce toxic interactions, and even death (21).

There are several routes for administering 5-MeO-DMT. This includes inhalation (~6–20 mg), intranasal (~10 mg), intravenous (~1–3 mg), sublingual (~10 mg), and oral (~30 mg) methods (18, 22). Inhalation by vapor is most commonly reported, given its accessibility and relative ease of use, particularly in naturalistic settings (6, 10). However, it can lead to intense rapid onset, relative to other dosage forms, like intramuscular injection. The onset, duration, and magnitude of subjective effects, occasioned by 5-MeO-DMT, are both route- and dose-dependent. For example, vaporization induces effects within ~10–15 s and peak experiences within ~2–5 min, resolving within ~25–30 min (6, 22, 23). Conversely, insufflation has a slower onset of action, due to delayed absorption, inducing effects within ~3–4 min and peak experiences within ~35–40 min, resolving within ~60–70 min (24). Irrespective of route, 5-MeO-DMT produces diverse subjective effects, including visual and auditory hallucinations, distorted time perception, and memory impairment (4). It also occasions peak mystical experiences comparable to high-dose psilocybin (25). Ego dissolution, a complete loss of self-identity, is frequently reported, as are profound near-death experiences (22, 25–28). 5-MeO-DMT can, therefore, be challenging to navigate, with reports of fear, extreme anxiety, and paranoia (29). Users also describe perceptual isolation, seeing “all white” or “all black” (30). This contrasts to classic psychedelics, like *N,N*-DMT and LSD, that produce highly detailed, complex mental imagery. From a clinical standpoint, 5-MeO-DMT shows signals of benefit to mental health and well-being (3, 4). However, there is a paucity of evidence in the field, particularly for trauma- and stress-related psychopathology.

Here, in accordance with CARE (CAse REport) guidelines (31), we present the first real-world, longitudinal case study on 5-MeO-DMT for post-traumatic stress disorder (PTSD). The subject provided written consent for publication and authorized disclosure of private health information. The data presented here were collected by the subject for their own interest and safety, and to monitor their progress over time. We then gained access to and analyzed the data retrospectively. To protect anonymity, the materials are not publicly available. This case study was exempt from ethics review and approval, in line with the Baylor College of Medicine Human Research Protections Manual, including Institutional Review Board procedures.

## Subject information

**Figure 1:**
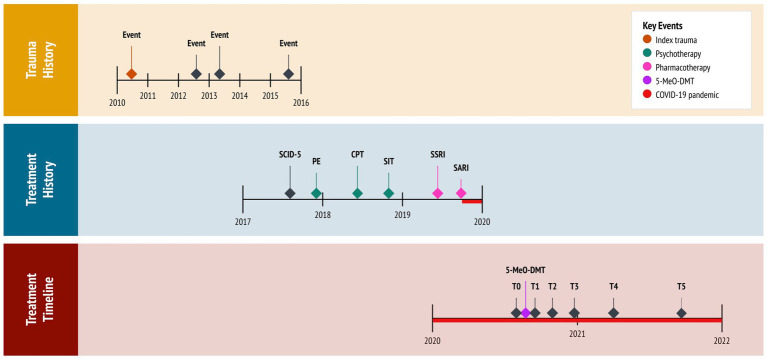
Timeline of historical and clinical events. 5-MeO-DMT, 5-methoxy-*N,N*-dimethyltryptamine (experimental treatment); COVID-19, coronavirus disease pandemic; CPT, cognitive processing therapy (cognitive behavioral therapy); PE, prolonged exposure (cognitive behavioral therapy); SARI, serotonin antagonist and reuptake inhibitor (trazadone); SCID-5, Structured Clinical Interview for DSM-5 (diagnostic assessment); SIT, stress inoculation therapy (cognitive behavioral therapy); SSRI, selective serotonin reuptake inhibitor (sertraline); T0, baseline; T1, 24 h follow-up; T2, 1 month follow-up; T3, 3 months follow-up; T4, 6 months follow-up; T5, 12 months follow-up.

A 23-year-old female presented with chronic refractory PTSD. She reported night terrors, trauma avoidance, negative affect, and hypervigilance. This developed from repeat sexual abuse, spanning six years as an adolescent. There was no relevant family history. Past interventions included variants of cognitive behavioral therapy (CBT), namely prolonged exposure (PE: 10 sessions), cognitive processing therapy (CPT: 12 sessions), and stress inoculation training (SIT: 8 sessions). These techniques targeted feared stimuli, maladaptive beliefs, and stress reactivity, respectively. However, each resulted in marginal improvements. She was then prescribed sertraline (Zoloft: 50 mg daily), a selective serotonin reuptake inhibitor (SSRI), following one week at 25 mg daily. This regimen adhered to pharmacotherapy guidelines for PTSD. Notwithstanding, the subject failed to respond adequately, reporting notable side effects, such as lethargy and disturbed sleep. She was, therefore, tapered off sertraline over the course of four weeks. This led to protracted symptoms and increased night terrors. Eventually, the subject was prescribed trazodone (Desyrel: 75 mg daily), a serotonin antagonist and reuptake inhibitor (SARI), for mixed insomnia. She experienced partial symptom relief and continued taking the medication, accordingly. The subject had no history of psychedelic use; however, she periodically smoked cannabis to manage her anxiety. See Figure 1 for a timeline of events.

The coronavirus (COVID-19) pandemic, restricting social contact with friends and family, further aggravated the subject’s condition. Critically, she desired to end “intense emotional pain” and “chronic sadness.” Isolated in lockdown, desperate for help, and at risk of suicide, the subject pursued self-treatment with 5-MeO-DMT. This was motivated by (1) her resistance to first- and second-line therapies for PTSD, having attempted multiple interventions; (2) evidence on the potential benefits of 5-MeO-DMT for anxiety and trauma, acquired from reading news articles and research studies; (3) new legislation approved in her state (Oregon, Measure 110), which decriminalized the possession of controlled substances, including psychedelics; and (4) access to a trauma-informed 5-MeO-DMT facilitator, to whom a friend referred her to.

## Diagnostic assessment

The subject was diagnosed with PTSD at 19 years of age. This was provided by her treating psychiatrist who, at the time, administered the Structured Clinical Interview for DSM-5 (SCID-5) (32), specifically the PTSD Module. The interview revealed a chronic course with severe PTSD symptoms and comorbid depression. Four years later, the subject pursued 5-MeO-DMT, independent from her psychiatrist, supported by a trauma-informed facilitator. The facilitator had extensive experience with 5-MeO-DMT, who advised on dosing and guided her experience. A licensed clinician, likewise, supported the subject in this pursuit. The clinician administered assessments, monitored her experience, and completed follow-ups. Assessments included the PTSD Checklist for DSM-5 [PCL-5; (33)], the Beck Hopelessness Scale [BHS; (34)] and the Clinical Global Impressions [CGI; (35)] questionnaire. These were used to track the subject’s progress over time, administered prior to 5-MeO-DMT dosing (i.e., at baseline), and again 24 h-, 1 month-, 3 months-, 6 months-, and 12 months later (i.e., at follow-ups). For safety purposes, the clinician took vital signs before, during, and after 5-MeO-DMT dosing. This consisted of blood pressure (mmHg), heart rate (bpm), and peripheral oxygen saturation (SpO_2_). To assess acute, subjective effects, the Mystical Experiences Questionnaire [MEQ-30; (36)] was administered 3 h post-dosing. The clinician observed the subject for a total of 5 h after her 5-MeO-DMT experience, and conducted follow-ups via phone 24, 36, and 72 h later, before switching to once a month.

### PTSD checklist for DSM-5

The PCL-5 is a 20-item measure of PTSD symptoms. It has excellent internal consistency (α = 0.94) (33), comprising four factors: ‘thought intrusion’, ‘stimuli avoidance’, ‘negative mood and cognitions’, and ‘altered reactivity’. Items are rated on a 5-point scale, with ‘not at all’ (0) and ‘extremely’ (4) as endpoints. The PCL-5 is scored by summing items within a given factor, as well as all items together. Total scores range from 0 to 80. Higher scores reflect greater symptom severity, with 31–33 typically used as the cut-off point for probabilistic PTSD. When monitoring symptoms, a 5–10-point difference indicates reliable change, not due to chance, whereas a 10–20-point difference indicates clinically significant change. The ‘past week’ version of the PCL-5 was utilized in this case study.

#### Impressions

At baseline, the subject’s total score was 72 of 80 (3.79 ± 0.42), meeting threshold criteria for ‘severe’ PTSD, and a provisional diagnosis. Regarding PCL-5 factors, she scored the highest on ‘altered reactivity’ (4.00 ± 0.00), followed by ‘thought intrusion’ (3.80 ± 0.45), ‘negative mood and cognitions’ (3.71 ± 0.49), and ‘stimuli avoidance’ (3.50 ± 0.71).

### Beck hopelessness scale

The BHS is a 20-item measure of hopelessness. It has excellent internal consistency (α = 0.97) (37), comprising three factors: ‘feelings about the future’, ‘loss of motivation’, and ‘future expectations’. Items are rated on a 2-point scale, using dichotomous ‘true’ (0/1) and ‘false’ (0/1) statements. The BHS is scored by summing items within a given factor, as well as all items together. Total scores range from 0 to 20. Higher scores reflect greater hopelessness, categorized into four levels: ‘normal’ (0–3), ‘mild’ (4–8), ‘moderate’ (9–14), and ‘severe’ (>14). A cut-off score of 9 is frequently used to detect risk of suicidal ideation and behavior.

#### Impressions

At baseline, the subject’s total score was 17 of 20 (0.85 ± 0.37), meeting threshold criteria for ‘severe’ hopelessness and suicide risk. Regarding BHS factors, she scored the highest on ‘feelings about the future’, (1.00 ± 0.00) and ‘future expectations’ (1.00 ± 0.00), followed by ‘loss of motivation’ (0.63 ± 0.52).

### Clinical global impressions

The CGI is a 3-item measure of global functioning. It was developed for clinical trials, aimed at capturing change after initiating a study drug. The CGI includes three factors. The first factor measures ‘illness severity’, rated on a 7-point scale, anchored by ‘normal and not at all ill’ (1) and ‘among the most extremely ill’ (7). The second factor measures ‘global improvement’, also rated on a 7-point scale, with ‘very much improved’ (1) and ‘very much worse’ (7) as endpoints. Finally, the third factor measures ‘therapeutic response’, rated on a 5-point scale, anchored by ‘marked improvement and no side effects’ (0) and ‘unchanged or worse and side effects outweigh therapeutic effect’ (4). This third factor considers both therapeutic efficacy and drug-related adverse events. A zero is allocated if there is no assessment. Each factor is rated separately, yielding no total scores.

#### Impressions

At baseline, the subject’s score for ‘illness severity’, regarding PTSD, was 6 of 7, meeting threshold criteria for ‘severely ill’. In particular, she exhibited disruptive trauma- and stress-related psychopathology, with symptoms considerably impairing her behavior and function. The other two factors, ‘global improvement’ and ‘therapeutic response’, were not assessed at baseline, as they measure changes after treatment.

## Therapeutic intervention

5-MeO-DMT was obtained and administered by the subject. The experience occurred in the comfort of her home. Guided by the facilitator, she first set an intention for the experience. “I want to understand and accept the roots of my trauma.” This was designed to help navigate potentially difficult psychedelic states and material, by re-centering the subject’s attention. Next, she engaged in body scan meditation, a specific form of mindfulness practice. This involved deep breathing and mind–body awareness, aimed at relaxation. The subject then inhaled 50 mg of vaporized bufotoxin, derived from the Sonoran Desert Toad (*Incilius alvarius*), slowly and consistently. This was estimated to contain 10–15 mg of 5-MeO-DMT [20–30% of total dried weight (38)]. Using a torch lighter, the bufotoxin was heated in a glass vial until its contents were vaporized. She held the dose for 10 s, exhaled slowly and consistently, and lied down with an eye mask on. Ambient music played in the background. The onset of effects was rapid (15–30 s), with peak effects lasting 10–15 min, resolving within 25–30 min. After the effects had subsided, the subject re-engaged in body scan meditation. She then discussed her experience with the facilitator, integrating newfound insights. Finally, the clinician reviewed the subject’s vital signs and asked about her experience, recording any undesirable reactions. Three hours later, the clinician administered the MEQ-30.

## Follow-up and outcomes

**Figure 2:**
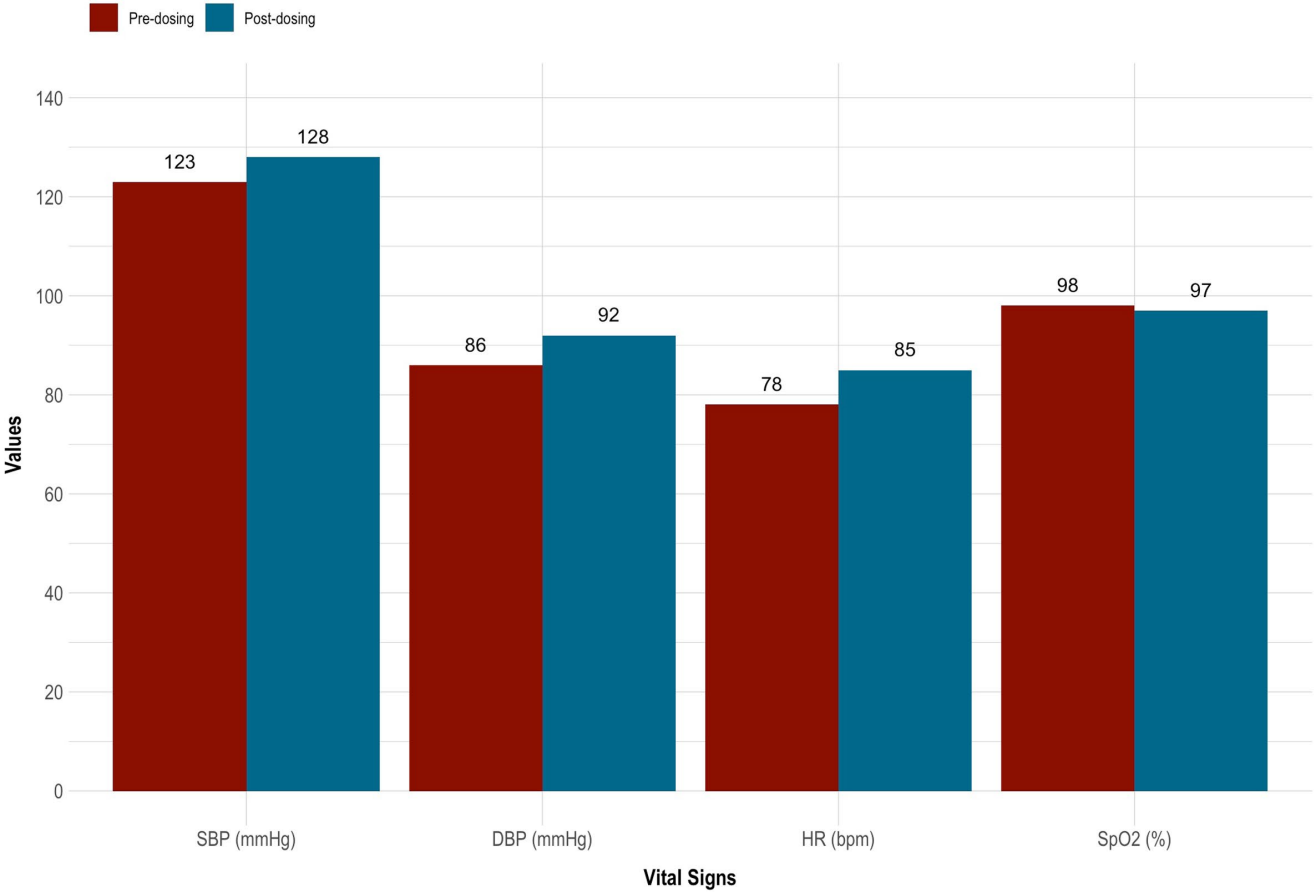
Vital signs taken before and after 5-MeO-DMT dosing. 5-MeO-DMT, 5-methoxy-*N,N*-dimethyltryptamine; DBP, diastolic blood pressure (mmHg); HR, heart rate (bmp); SBP, systolic blood pressure (mmHg); SpO_2_, peripheral blood oxygenation (%).

5-MeO-DMT was generally tolerated by the subject. Mild nausea was reported, which resolved within 30 min. There were slight increases in systolic blood pressure (126.00 ± 3.54), diastolic blood pressure (89.00 ± 4.24), and heart rate (81.50 ± 4.95), whereas oxygen saturation (97.50 ± 0.71) remained stable. See Table 1 and Figure 2. Overall, no drug-related, serious adverse events occurred. However, the subject reported “profoundly strong” subjective effects. She described being “instantly blasted” into another dimension. At first, colors were extremely vivid, then morphed into “complete whiteness.” The subject failed to make sense of psychedelic content, stating that visuals were “bright and god-like,” yet vague and fleeting. She also reported increased body temperature and euphoria. “I felt really warm, like my body was melting. It was calm and blissful.” This was accompanied by radical ego dissolution. “I had no identity. I was still alive, but my body was gone. It was quite overwhelming. I just had to surrender.”

**Table 1:** Clinician-reported vital signs.

Pre-dosing				Post-dosing				Difference			
SBP	DBP	HR	SpO_2_	SBP	DBP	HR	SpO_2_	SBP	DBP	HR	SpO_2_
123	86	78	98	128	92	85	97	5	6	7	–1

DBP, diastolic blood pressure (mmHg); HR, heart rate (bmp); SBP, systolic blood pressure (mmHg); SpO_2_, oxygen saturation (%).

**Figure 3:**
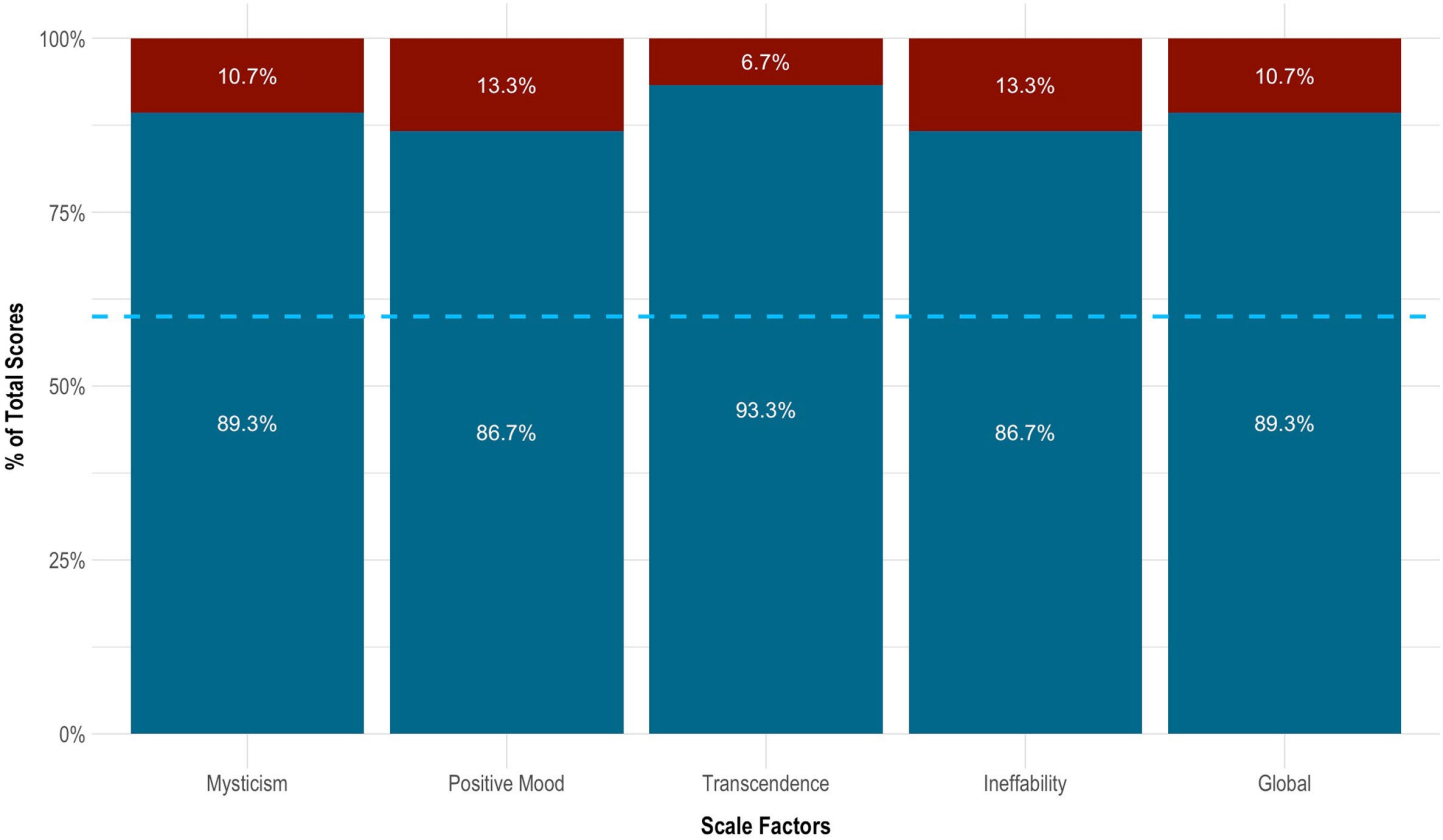
Mystical effects of 5-MeO-DMT. Blue dotted line indicates the cut-off point for a complete mystical experience (≥60% of total scores across factors). 5-MeO-DMT, 5-methoxy-*N,N*-dimethyltryptamine; MEQ-30, Mystical Experience Questionnaire.

On the MEQ-30, the subject endorsed strong mystical-like effects. Her total score was 135 of 150 (4.47 ± 0.62). She also met criteria for a ‘complete mystical experience’. This was evidenced by scoring ≥60% of the maximum possible scores on all four factors of the MEQ-30: ‘mysticism’ (4.47 ± 0.62 [89.3%]), ‘positive mood’ (4.33 ± 0.75 [86.7%]), ‘transcendence’ (4.67 ± 0.47 [93.3%]), and ‘ineffability; (4.33 ± 0.47 [86.7%]). See Figure 3 for details.

**Figure 4:**
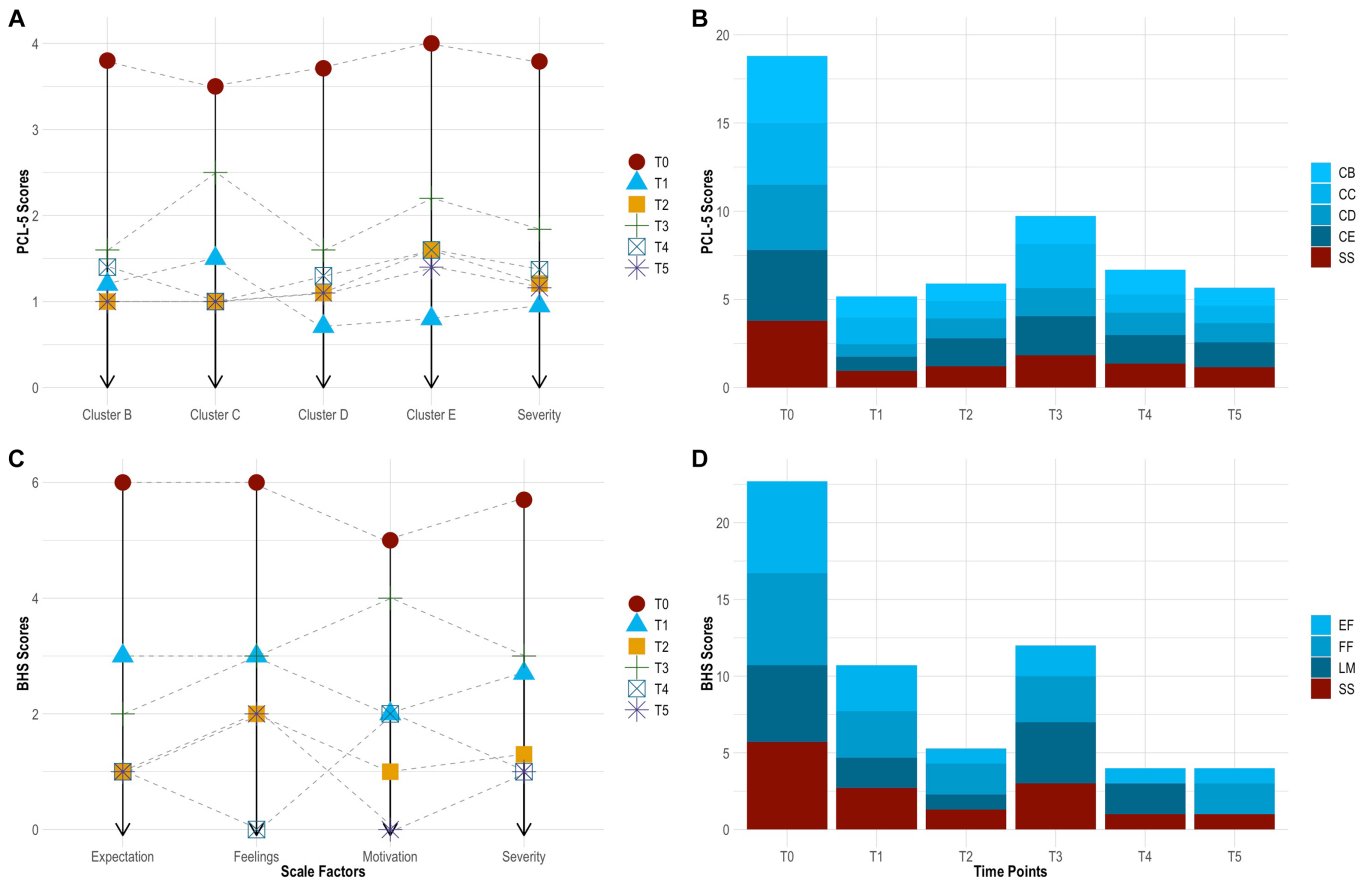
Change in PTSD symptoms by time point [**(A)**, line graph]. Change in PTSD symptoms across time [**(B)**, bar chart]. Change in hopelessness symptoms by time point [**(C)** line graph]. Change in hopelessness symptoms across time [**(D)**, bar chart]. BHS, Beck Hopelessness Scale; CB, cluster B (thought intrusion); CC, cluster C (stimuli avoidance); CD, cluster D (negative mood and cognitions); CE, cluster E (altered reactivity); EF, expectations about the future (hopelessness); FF, feelings about the future (hopelessness); LM, loss of motivation (hopelessness); PCL-5, PTSD Checklist for DSM-5; PTSD, post-traumatic stress disorder; SS, symptom severity; T0, baseline; T1, 24 h follow-up; T2, 1 month follow-up; T3, 3 months follow-up; T4, 6 months follow-up; T5, 12 months follow-up.

On the PCL-5, the subject had a clinically significant change in PTSD, which sustained across time. This was evidenced by a ≥ 10-point reduction in total scores from baseline to 24 h (−54 points), 1 month (−49 points), 3 months (−37 points), 6 months (−46 points), and 12 months (−50 points) follow-up. In particular, her symptoms decreased by 75.0% from baseline to 24 h (3.79 ± 0.42 vs. 0.95 ± 0.71), increased by 27.8% from 24 h to 1 month (0.95 ± 0.71 vs. 1.21 ± 0.63), increased by 52.2% from 1 month to 3 months (1.21 ± 0.63 vs. 1.84 ± 0.76), decreased by 25.7% from 3 months to 6 months (1.84 ± 0.76 vs. 1.37 ± 0.68), and finally decreased by 15.4% from 6 months to 12 months (1.37 ± 0.68 vs. 1.16 ± 0.60) follow-up. From baseline to 12 months follow-up, she experienced the greatest improvement in ‘negative mood and cognitions’ (3.71 ± 0.49 vs. 1.10 ± 0.69), followed by ‘thought intrusion’ (3.80 ± 0.45 vs. 1.00 ± 0.71), ‘altered reactivity’ (4.00 ± 0.00 vs. 1.40 ± 0.55), and ‘stimuli avoidance’ (3.50 ± 0.71 vs. 1.00 ± 0.00). See Table 2 and Figures 4A,B.

**Table 2:** Self-reported outcome measures.

Measures	T0	T1	T2	T3	T4	T5
PCL-5 Factors	Baseline	24 Hours	1 Month	3 Months	6 Months	12 Months
Thought Intrusion	19 (3.80 ± 0.45)	6 (1.20 ± 0.84)	5 (1.00 ± 0.71)	8 (1.60 ± 0.89)	7 (1.40 ± 0.55)	5 (1.00 ± 0.71)
Stimuli Avoidance	7 (3.50 ± 0.71)	3 (1.50 ± 0.71)	2 (1.00 ± 0.00)	5 (2.50 ± 0.71)	2 (1.00 ± 1.41)	2 (1.00 ± 0.00)
Negative Concept	26 (3.71 ± 0.49)	5 (0.71 ± 0.76)	8 (1.10 ± 0.69)	11 (1.60 ± 0.53)	9 (1.30 ± 0.49)	8 (1.10 ± 0.69)
Altered Reactivity	20 (4.00 ± 0.00)	4 (0.80 ± 0.45)	8 (1.60 ± 0.55)	11 (2.20 ± 0.84)	8 (1.60 ± 0.89)	7 (1.40 ± 0.55)
Symptom Severity	72 (3.79 ± 0.42)	18 (0.95 ± 0.71)	23 (1.21 ± 0.63)	35 (1.84 ± 0.76)	26 (1.37 ± 0.68)	22 (1.16 ± 0.60)
BHS Factors	Baseline	24 Hours	1 Month	3 Months	6 Months	12 Months
Future Feelings	6 (1.00 ± 0.00)	3 (0.50 ± 0.55)	2 (0.33 ± 0.52)	3 (0.50 ± 0.55)	0 (0.00 ± 0.00)	2 (0.33 ± 0.52)
Loss of Motivation	5 (0.63 ± 0.52)	2 (0.25 ± 0.46)	1 (0.13 ± 0.35)	4 (0.50 ± 0.53)	2 (0.25 ± 0.46)	0 (0.00 ± 0.00)
Future Expectations	6 (1.00 ± 0.00)	3 (0.50 ± 0.55)	1 (0.17 ± 0.41)	2 (0.33 ± 0.52)	1 (0.17 ± 0.41)	1 (0.17 ± 0.41)
Symptom Severity	17 (0.85 ± 0.37)	8 (0.40 ± 0.50)	4 (0.20 ± 0.41)	9 (0.45 ± 0.51)	3 (0.15 ± 0.37)	3 (0.15 ± 0.37)

BHS, Beck Hopelessness Scale; PCL-5; PTSD Checklist for DSM-5; T0, baseline; T1, 24 h follow-up; T2, 1 month follow-up; T3, 3 months follow-up; T4, 6 months follow-up; T5, 12 months follow-up.

On the BHS, the subject showed robust improvement in hopelessness. This also sustained across time. Her symptoms decreased by 52.9% from baseline to 24 h (0.85 ± 0.37 vs. 0.40 ± 0.50), decreased by 50.0% from 24 h to 1 month (0.40 ± 0.50 vs. 0.20 ± 0.41), increased by 125.0% from 1 month to 3 months (0.40 ± 0.50 vs. 0.45 ± 0.51), decreased by 66.7% from 3 months to 6 months (0.45 ± 0.51 vs. 0.15 ± 0.37), and remained stable from 6 months to 12 months (0.15 ± 0.37 vs. 0.15 ± 0.37) follow-up. From baseline to 12 months follow-up, she experienced the greatest improvement in ‘loss of motivation’ (0.63 ± 0.52 vs. 0.00 ± 0.00) and ‘future expectations’ (1.00 ± 0.00 vs. 0.17 ± 0.41), followed by ‘feelings about the future’ (0.63 ± 0.52 vs. 0.33 ± 0.52). Further, the subject had a clinically significant change in suicide risk. This was evidence by scoring ≤9 at 24 h (score = 8), 1 month (score = 4), 3 months (score = 9), 6 months (score = 3), and 12 months (score = 8) follow-up. See Table 2 and Figures 4C,D.

On the CGI, the clinician reported marked reductions in PTSD, which sustained across time. Rated at each time point, she presented as ‘severely ill’ at baseline (score = 6), ‘mildly ill’ at 24 h (score = 3), ‘borderline ill’ at 1 month (score = 2), ‘mildly ill’ at 3 months (score = 3), ‘not at all ill’ at 6 months (score = 1), and ‘not at all ill’ at 12 months (score = 1) follow-up. These ratings considered the clinician’s total experience treating PTSD. Relative to baseline, the subject’s global functioning changed from ‘much improved’ at 24 h post-dosing (score = 2), representing a significant change, with increased functioning and moderate symptoms, to ‘very much improved’ at 12 months follow-up (score = 1), indicating a substantial change, with good functioning and minimal symptoms. This was judged independent from any beliefs about 5-MeO-DMT. Finally, based on drug effect, her therapeutic response was ‘marked’ at 24 h post-dosing (score = 2), with side effects that did not significantly interfere with functioning, and ‘marked’ again at 12 months-follow-up (score = 1), with no side effects. See Table 3.

**Table 3:** Clinician-reported outcome measures.

Measures	T0	T1	T2	T3	T4	T5
CGI Factors	Baseline	24 Hours	1 Month	3 Months	6 Months	12 Months
Severity of Illness	6.0	3.0	2.0	3.0	1.0	1.0
Global Improvement	—	2.0	1.0	3.0	1.0	1.0
Therapeutic Response	—	2.0	1.0	1.0	1.0	1.0

CGI, Clinical Global Impressions; T0, baseline; T1, 24 h follow-up; T2, 1 month follow-up; T3, 3 months follow-up; T4, 6 months follow-up; T5, 12 months follow-up. Global improvement and therapeutic response were not assessed at baseline, as they measured post-dosing changes only.

## Discussion

In this case study, a single dose of vaporized toad bufotoxin, containing 5-MeO-DMT, led to clinically significant improvements in PTSD, with next-day effects. These gains were sustained at 1-, 3-, 6-, and 12-months follow-up. Moreover, the subject showed striking reductions in hopelessness and related suicide risk. These changes were, likewise, durable across time. Self-reported improvements further reflected clinician-observed changes in global functioning. 5-MeO-DMT was generally tolerated. No drug-related, serious adverse events occurred. However, there were nominal increases in blood pressure and heart rate. This did not extend to oxygen saturation. Subjective effects were also overwhelming. Interestingly, 5-MeO-DMT produced more visual content than previously described (30). Colors appeared at the beginning of her experience, then faded into transcendent light; the latter being more consistent with literature (30). The subject’s dose and setting likely impacted her perceptual experience (38). Regardless, more data is needed to characterize the phenomenology of 5-MeO-DMT, and how this compares to other psychedelics. This is particularly important for optimizing facilitation and harm reduction practices, in helping patients navigate psychedelic states, as well as for targeting PTSD and chronic stress pathology.

Furthermore, the subject endorsed a strong and complete mystical experience. While the mechanism underlying her therapeutic response is unknown, it may be explained, in part, by the epistemological or ‘noetic quality’ of mystical states, occasioned by 5-MeO-DMT (39). These psychological states are characteristic of psychedelics, namely serotonergic compounds (39); have been shown to correlate, mediate, and predict therapeutic efficacy (40); and include feelings of transcendence, ego dissolution, and ineffability as well as unity, love, and peace (41). Thus, people have rated mystical experiences in their top five most important life events, in terms of personal meaning and spiritual significance, next to giving birth or losing a loved one (42, 43). These effects can persist up to 30 years after taking a psychedelic (44). In the present case study, the subject described the mystical effects of 5-MeO-DMT as both substantial and enduring. “It was the most profound and frightening experience of my life. I saw bright colors. I was connected to all things. I disappeared into space. I smiled for the first time in a long time. I cried and screamed. I forgot about [my] pain and trauma… then relived it. My body had permission to heal. I moved on. It’s hard to put it into words… beautiful and challenging I guess…feeling everything and nothing at once. But it allowed me to view my trauma in a different way. Like a superpower. That insight has stayed with me.” Other possible mechanisms of change, from a psychological standpoint, include re-processing and transforming traumatic material.

This case study aligns with previous findings in the literature. For instance, in a retrospective, epidemiology survey on 5-MeO-DMT (*n* = 515; *M*_age_ = 35.4; male = 79%), 79% of participants with psychiatric disorders reported improved PTSD following 5-MeO-DMT use (22). Most participants (90%) had moderate-to-strong mystical experiences, while a significant proportion (37%) had challenging ones. In another retrospective, international survey (*n* = 99; *M*_age_ = 37.4; male = 74%), 79% of participants with past or present PTSD, who had used 5-MeO-DMT at least once in their lifetime, reported improved symptomatology (45). They also endorsed significantly stronger mystical experiences than those who did not experience symptom improvement or regressed. Most recently, Davis et al. (46) examined ibogaine and 5-MeO-DMT for trauma-related psychological and cognitive impairment, specifically among U.S. Special Operations Forces Veterans (*n* = 51; *M*_age_ = 40.0; male = 96%). They analyzed retrospective data collected 30 days before and 30 days after a clinical psychedelic program in Mexico. The results showed significant and large reductions in depression, suicidal ideation, anxiety, PTSD, and cognitive impairment. Participants additionally reported increased psychological flexibility, which was strongly associated with improvements in all constructs, excluding suicidality.

Other studies have investigated 5-MeO-DMT in naturalistic settings. For example, in an observational group study, using structured dosing protocols, researchers examined clinical correlates of 5-MeO-DMT (27). Among healthy participants (*n* = 362; *M*_age_ = 47.7; male = 55%), 80% with depression and 79% with anxiety reported spontaneous, unintended reductions in symptoms. This was associated with stronger mystical experiences, as well as higher ratings of spirituality and meaning in life. In another observational study, Uthaug et al. (47) investigated sub-acute and long-term effects of 5-MeO-DMT on affect and cognition. Among healthy participants (*n* = 42; *M*_age_ = 38.0; male = 60%), ratings of depression, anxiety, and stress decreased 24 h post-intake and reached significance at 4 weeks follow-up. Those who experienced high levels of ego dissolution or oceanic boundlessness, two markers of a mystical experience, displayed lower levels of depression and stress. However, this did not extend to anxiety.

Of note, the subject partially regressed at 3-months follow-up. She reported new onset of night terrors, the nature of which could not be recalled. These night terrors reflect higher scores across all measures at this time point, relative to the others. A phenomenon known as ‘reactivation’, similar to flashbacks, is commonly reported by 5-MeO-DMT users (22, 48). This involves re-experiencing parts of a drug-induced state post-administration, which can occur days, weeks, or even months later (49, 50). Additionally, the probability of 5-MeO-DMT reactivation increases with being female, dosing in a structured group format, and having a stronger mystical experience (48). All three of these factors applied to this case study. As such, the subject may have endured a reactivation event following 5-MeO-DMT, presenting as negatively-valenced night terrors. Alternatively, the benefits of 5-MeO-DMT may have only lasted for three months. Despite the partial regression, scores across all measures remained below clinical thresholds, with symptoms naturally remitting overtime. The onset of night terrors was not considered a ‘serious adverse event’, as its association with 5-MeO-DMT could not be definitively concluded. It neither was life-threatening, required intervention or hospitalization, resulted in persistent or significant disability, nor led to the subject’s death.

## Strengths and limitations

The longitudinal nature of this case study serves as its primary strength, with repeated observations collected over a 1-year period. Findings are more robust, given the subject’s treatment resistance and disease chronicity, the complexity of this clinical population, and the limitation in available effective, evidence-based interventions. Further, the presence of psychiatric comorbidities, the lack of polypharmacy or medication washout, and the naturalistic setting better reflect patients in the real world. The use of well-validated measures, capturing both subject- and clinician-reported changes, is an additional strength. Notwithstanding, this case study is inherently limited.

First, it describes the presentation, treatment, and follow-up of a single person. Hence, the results cannot be generalized to others with PTSD. Second, the dose of 5-MeO-DMT was estimated by the subject, based on visual inspection. The precise amount cannot be determined, accordingly. Third, the source for obtaining toad bufotoxin, containing the 5-MeO-DMT, is unknown. The compound’s integrity may have been compromised as a result. Fourth, 5-MeO-DMT was self-administered by the subject. This is not considered a suitable clinical or pharmaceutical application, primarily due to safety reasons. Finally, there is no evidence that 5-MeO-DMT, in and of itself, produced therapeutic activity reported in this case study. Facilitation practices, like body scan meditation, for instance, may have confounded the results, magnifying or diminishing therapeutic effects. Findings should, therefore, be interpreted with caution, and only serve to catalyze future research. This is particularly important, as the field is far from establishing clinical efficacy, real-world effectiveness, and standard treatment protocols for 5-MeO-DMT in PTSD and beyond. Additionally, naturalistic psychedelic use has steadily increased over the past decade (51). This is likely due to media coverage, advances in research, and changes to legislation. It is, thus, critical to balance discussions on 5-MeO-DMT and other psychedelics with clear and careful acknowledgement of safety risks.

Looking ahead, the next logical step is to conduct pilot studies that explore the safety, tolerability, and preliminary efficacy of 5-MeO-DMT for PTSD, in larger and more diverse samples. Including a richer battery of psychometric instruments is highly encouraged. Results could then inform open-label, randomized, and adaptive trials to further characterize 5-MeO-DMT for this patient population; and to explore different therapeutic approaches, including adjunctive psychotherapy, which may augment patient adherence and therapeutic outcomes. Incorporating moderated mediation models, as statistical analyses, is also encouraged in future work. This would allow researchers to control for covariates, like age and gender, while examining potential underlying mechanisms, such as mystical experiences.

## Conclusion

This case study is the first to report the longitudinal effects of 5-MeO-DMT for chronic refractory PTSD, complicated by hopelessness and suicidality. The results showed that 5-MeO-DMT offered fast-acting, robust, and sustained improvements in symptomatology, and was generally tolerable and safe to administer. However, this was not without risks, as evidenced by acute nausea, overwhelming subjective effects, and late onset of night terrors. Further research is warranted to replicate and extend these findings, which are inherently limited, non-generalizable, and rely on methods not clinically accepted. This can be achieved through clinical and naturalistic studies, in controlled and uncontrolled environments, to effectively converge on safety, efficacy, effectiveness, and durability of 5-MeO-DMT for PTSD. Evidence can then be leveraged to optimize therapeutic delivery, as well as develop standard clinical practice guidelines.

## Data availability statement

The original contributions presented in the study are included in the article/supplementary material, further inquiries can be directed to the corresponding author.

## Ethics statement

Ethical approval was not required for the study involving humans in accordance with the local legislation and institutional requirements. Written informed consent to participate in this study was not required from the participants or the participants’ legal guardians/next of kin in accordance with the national legislation and the institutional requirements. Written informed consent was obtained from the individual(s) for the publication of any potentially identifiable images or data included in this article.

## Author contributions

AR: Conceptualization, Formal analysis, Project administration, Visualization, Writing – original draft. RK: Investigation, Writing – original draft, Writing – review & editing. PS: Conceptualization, Funding acquisition, Project administration, Supervision, Writing – review & editing. LB: Writing – original draft, Writing – review & editing. RA: Writing – original draft, Writing – review & editing. MK: Writing – original draft, Writing – review & editing. NB: Validation, Writing – review & editing. LJ: Validation, Writing – review & editing. MG: Validation, Writing – review & editing. JB: Supervision, Validation, Writing – review & editing. LA: Conceptualization, Supervision, Validation, Writing – review & editing.
